# Epigenetic Upregulation of HGF and c-Met Drives Metastasis in Hepatocellular Carcinoma

**DOI:** 10.1371/journal.pone.0063765

**Published:** 2013-05-28

**Authors:** Olorunseun O. Ogunwobi, William Puszyk, Hui-Jia Dong, Chen Liu

**Affiliations:** 1 Department of Pathology, Immunology and Laboratory Medicine, University of Florida, Gainesville, Florida, United States of America; 2 Shands Cancer Center, University of Florida, Gainesville, Florida, United States of America; University of North Carolina School of Medicine, United States of America

## Abstract

Hepatocyte growth factor (HGF) and its receptor, c-Met, are important regulators of growth and differentiation of healthy hepatocytes. However, upregulation of HGF and c-Met have been associated with tumor progression and metastasis in hepatocellular carcinoma (HCC). Hematogenous dissemination is the most common route for cancer metastasis, but the role of HGF and c-Met in circulating tumor cells (CTCs) is unknown. We have isolated and established a circulating tumor cell line from the peripheral blood of a mouse HCC model. Our studies show that these CTCs have increased expression of HGF and c-Met in comparison to the primary tumor cells. The CTCs display phenotypic evidence of epithelial-mesenchymal transition (EMT) and the EMT appears to be inducible by HGF. Epigenetic analysis of the c-Met promoter identified significant loss of DNA methylation in CTCs which correlated with overexpression of c-Met and increased expression of HGF. Six specific CpG sites of c-Met promoter demethylation were identified. CTCs show significantly increased tumorigenicity and metastatic potential in a novel orthotopic syngeneic model of metastatic HCC. We conclude that during hematogenous dissemination in HCC, CTCs undergo EMT under the influence of increased HGF. This process also involves up regulation of c-Met via promoter demethylation at 6 CpG sites. Consequently, targeting HGF and c-Met expression by CTCs may be a novel non-invasive approach with potential clinical applications in HCC management.

## Introduction

Over 90% of mortality from cancer is due to metastatic spread [1]. In the majority of cancer patients, the primary tumor is unlikely to kill whereas the metastatic disease will result in mortality. Unfortunately, while significant progress has been made in understanding the etiology and progression of many primary cancers, the basis for metastases of cancers remains largely unclear.

The importance of understanding the biological basis of cancer metastasis has generated interest in this area of research and has led to the proposition of a number of biological concepts as potential mechanisms in cancer metastasis. One concept is that cancer cells undergo epithelial-mesenchymal transition (EMT) in order to acquire metastatic ability [2], [3]. Although many *in vitro* and animal studies have provided experimental support for this idea, some studies have questioned the usefulness of this concept in explaining cancer metastasis [4], [5]. Consequently, there remains an urgent need to clarify the exact role of EMT in cancer metastasis.

Previous research has focused on the biology of cancer cells from the primary tumor and cancer cells from metastatic lesions. The processes which enable cancer cells to escape from their primary site and allow them to survive in the immunologically hostile environment of blood and acquire the capability to colonize secondary sites are largely unknown. We hypothesized that viable cancer cells able to circulate in the blood of cancer patients possess important molecular and functional features that are different from cancer cells at the primary site of tumor. These differences may account for the metastatic capability of these cells. This approach to the study of cancer metastasis is important because distant metastases and tumor self-seeding are believed to occur almost entirely via hematogenous spread [6]–[8]. Moreover, it has potential to reveal novel insights into the mechanisms of cancer metastasis and can be optimized for clinical use for personalized cancer management [9], [10].

Previous examination of the blood of cancer patients have largely employed relatively controversial methods that are generally irreproducible and reveal only limited information regarding the existence of circulating tumor cells (CTCs) [11]–[14]. In using these methods, investigators have frequently assumed that all CTCs are CD45 negative and cytokeratin and EpCam positive [11]. This assumption is questionable since it would result in the identification of only epithelial cells whereas there is significant data suggesting that acquisition of mesenchymal characteristics is a phenomenon that takes place in cancer [2], [15]. Some methods for detection of CTCs have also used tumor-specific markers [16], [17], real-time *in vivo* imaging [18] and microfluidic-based systems [19]. Importantly, none of these methods has been used in whole animal models to successfully establish CTC lines that can be used for detailed molecular and functional characterization of CTC biology in a reproducible and consistent manner. Accomplishing this will be needed to definitively clarify the exact functional role of CTCs in cancer metastasis.

Here, for the first time, we report elucidation of the functional role and regulatory mechanisms of HGF and its receptor, c-Met, in CTC biology during hematogenous metastasis of HCC. These data are based on novel CTC lines and a novel syngeneic orthotopic metastatic HCC model. These novel models are derived from a highly reproducible method for the isolation and culture of viable CTCs from whole blood samples of a syngeneic murine hepatocellular carcinoma (HCC) model.

## Materials and Methods

### Ethics Statement

All animal studies were approved by the University of Florida Institutional Animal Care and Use Committee (IACUC): protocol number 201106529.

### Cell culture and reagents

The BNL 1ME A.7R.1 murine HCC cell line was obtained from the American Type Culture Collection (ATCC) and has been previously described [20]. OL0825, OL2548 and OL2549 are novel blood-derived CTC lines established during this study and described in the results section of this paper. All cell lines were maintained in culture in DMEM supplemented with 10% fetal bovine serum (FBS), 2 mM L-glutamine, non-essential amino acids, 100 mg/l penicillin and 100 mg/l streptomycin. Cells were incubated at 37°C in a humidified incubator with air and CO_2_. All cell culture reagents were obtained from Cellgro (Manassas, VA). 5-aza-2′-deoxycytidine (AZA) was obtained from Sigma (St Louis, MO).

### Subcutaneous implantation of tumor cells

Following University of Florida IACUC-approved protocols, 1×10^6^ BNL 1ME A.7R.1 cells or OL0825 cells or OL2548 cells or OL2549 cells suspended in phosphate buffered saline were implanted subcutaneously into the flank of Balb/c mice as previously described [20].

### Survival surgical implantation of tumor cells

Following University of Florida IACUC-approved protocols, Balb/c mice were anesthetized by isoflurane inhalation. Their abdomen was shaved and sterilized. Mice were draped, incision site exposed and surgical site cleaned. A small incision was made on the abdomen just below the xyphoid process. The liver was exposed and 1×10^6^ BNL 1ME A.7R.1 or OL0825 cells suspended in phosphate buffered saline were injected into the liver. The liver was then carefully placed back into the abdominal cavity. The incision site was closed and mice were monitored until stable and then placed back in their regular accommodation. Mice were humanely euthanized as soon as clinical evidence of tumor development was observed (reduced body condition score as detailed in IACUC-approved protocol).

### Establishment of novel blood-derived circulating tumor cell lines

500–1000 μl whole blood was collected via the intracardiac route from Balb/c mice that developed tumors from subcutaneous and survival surgical intrahepatic implantation with 1×10^6^ BNL 1ME A.7R.1 HCC cells following University of Florida IACUC-approved protocols. After centrifugation, the buffy coat layers were collected and subjected to red blood cell lysis. Lysed samples were washed in PBS and seeded into cell culture medium containing 10% fetal bovine serum, 1% penicillin, 1% streptomycin and cells were incubated at 37°C in a humidified incubator with air and CO_2_. Proliferation of tumor cells was observed within 2–7 days. This ultimately led to the establishment of the novel CTC lines OL0825, OL2548 and OL2549.

### Verification of source of novel blood-derived circulating tumor cell lines

There is currently no commercially available method for the verification of mouse cell lines. Therefore, a novel polymerase chain reaction (PCR)-based method that amplifies only one specific DNA segment of the mouse β-globin gene was used to verify that the novel established circulating tumor cell lines (OL0825, OL2548 and OL2549) are mouse cells just like the originally implanted BNL 1ME A.7R.1 HCC line. This method was originally described and validated by Steube *et al* [21]. Briefly, genomic DNA was isolated from all cell lines using the High Pure PCR Template Preparation Kit (Roche, IN, USA). PCR amplification of one specific DNA segment of the mouse β-globin gene was performed using specific primers. Primer sequences are listed in Table S1. The PCR reaction utilized the TaKaRA hot start polymerase and 400ng of genomic DNA. Distilled water and genomic DNA from the human Huh7 HCC cell line were used as negative controls to verify specificity and accuracy of the assay. Furthermore, BNL 1ME A.7R.1, OL0825, OL2548 and OL2549 are the only mouse cell lines currently cultured in our laboratory. The PCR protocol used was: two minutes at 94°C, three minutes at 72°C, 35 cycles of denaturation at 94°C for 30 seconds, annealing at 61.6°C for 30 seconds and extension at 72°C for one minute. PCR products were visualized after electrophoresis on a 1.2% ethidium bromide-stained agarose gel. In addition, to verify that the novel established circulating tumor cell lines (OL0825, OL2548 and OL2549) are hepatocytes just like the originally implanted BNL 1ME A.7R.1 HCC line, we performed immunostaining for the hepatocyte-specific marker cAMP responsive element binding protein 3-like 3 (CREB3L3) using a specific antibody (sc-292135, Santa Cruz Biotechnology, CA) and our previously published protocol [22].

### Real-time quantitative PCR (qPCR)

qPCR was performed as previously described [20]. The primers used for amplification of HGF, c-Met, E-cadherin, vimentin, collagen I and fibronectin have also been previously described [20]. qPCR was performed using SYBR Green. Reactions were conducted in a 96-well spectrofluorometric thermal cycler (Applied Biosystems, CA).

### Western blotting

Cells were cultured in a monolayer until 60%–70% confluent. Immunoblotting was performed as previously described [23]. The specific primary antibodies used for detection of E-cadherin, fibronectin, collagen I, vimentin and actin were recently described [20]. HGF (sc-13087), c-Met (sc-161) and phosphorylated c-Met (sc-34085) expressions were detected using primary antibodies obtained from Santa Cruz Biotechnology (Santa Cruz, CA). Immunoreactive proteins were visualized by incubating in HRP-conjugated secondary antibodies. Chemiluminescence was detected by incubating in an equal-parts mixture of the SuperSignal West Pico stable peroxide solution and luminol/enhancer solution (Pierce, IL) and subsequently using an image processing machine. Densitometry was performed using the Image J software (NIH, MD).

### Enzyme-linked immunosorbent assay (ELISA)

Cells were seeded at 1×10^6^ cells per well in 6-well plates and cultured in complete medium for 48 hours. Analysis of HGF released into serum-free medium over 24 hours was performed using a specific mouse HGF ELISA (R&D Systems) as previously described [20].

### Hematoxylin and Eosin staining

Hematoxylin and eosin staining of tissue specimens was performed by our Molecular Pathology Core lab following standard operating procedures. Stained slides were visualized with a microscope equipped with imaging software. Images were reviewed independently by 2 liver pathologists (Chen Liu, MD, PhD and Elaine Salazar, MD).

### DNA methylation analysis

In order to identify the mechanism behind the increase in expression of HGF and c-Met in OL0825 in comparison to BNL 1ME A.7R.1 cells, the DNA methylation pattern of promoters for c-Met and HGF were analyzed by high resolution melt (HRM) analysis. A number of primers were designed to tile over the promoter and first exon of each gene (see list in Table S3). Primers for HGF overlap a GC rich region proximal to the promoter. Primers for c-Met overlap with the CpG island proximal to the transcription initiation site. Primers were either designed to be bisulfite-specific or optimized for high resolution analysis [24]. Primers were optimized using commercially prepared; highly methylated and unmethylated genomic DNA samples (EpigenDx, MA, USA) as standards for bisulfite conversion. Primers were optimized using MgCl_2_ and temperature gradients to identify optimal conditions for both amplification and melt analysis. Unconverted genomic DNA was used as a control to ensure that primers only amplified bisulfite converted DNA. Two primer pairs, *c-Met*_F2 and HGF_F3 were found to be most optimal for high resolution melt analysis using commercial control DNA samples and then used to analyze the respective CpG Islands of BNL 1ME A.7R.1 and OL0825 cells. DNA was extracted from cultured cells using the Qiagen blood and tissue kit. Approximately 2 million cells were used per reaction. DNA was eluted in 100 µL of buffer AE and quantified using the nanovue spectrophotometer. 1 µg of DNA from each DNA sample was bisulfite converted. DNA was collected from BNL 1ME A.7R.1 and OL0825 cells. DNA samples were bisulfite converted using a modified protocol [25]. Each sample was eluted in 30 µl and each bisulfite-specific PCR used 2 µl of DNA.

PCRs were performed in triplicate using RT^2^ SYBR green mix (Qiagen) on the Step one plus Real time PCR platform (Applied Biosystems). After preliminary analysis using HRM analysis the reverse primer of each primer pair was re-designed to include a 5′ biotin bead, to tag PCR products for pyrosequencing. Additional sequencing primers were designed to sequence CpGs in the amplicon and to give a quantifiable read of methylation at single CpG resolution. Pyrosequencing was performed using 2 sequencing primers for HGF_F3 providing sequence for 3 CpGs. Pyrosequencing was performed for c-Met_F2 using 1 sequencing primer providing sequence information on 9 CpGs.

### Pyrosequencing

Pyrosequencing analysis was by Pyrosequencer ID (Qiagen, Inc., Valencia, CA). Detection of HGF and c-Met gene methylation status were achieved by quantitative measure of methylated (C)/un-methylated (T) allele peak ratios by the pyrosequencer. The assay was designed for detecting c-Met (analyzed 9 CpG sites) at chromosome 6 at positions 17413351-17413473 and HGF (analyzed 2 CpG sites) at chromosome 5 at positions 16059123-16059304. Sequence accessions match to the UCSC Genome Browser Mouse assembly (NCBI37/mm9), July 2007. Briefly, the specific test procedure is described as follows: 25 µL of biotinylated PCR products are immobilized on streptavidin coated Sepharose beads (Streptavidin Sepharose High Performance, GE Healthcare). The mixtures were incubated at room temperature for 5 minutes, and then the beads that contain PCR products are cleaned, denatured, and washed. Sequencing primers are annealed to the single strand DNA fragments. The sequence reaction and the detection are performed by pyrosequencing following the manufacturer's protocol. The allelic methylation calculation was performed by PyroMark ID Pyro Q_CpG_1.0.9. The results are reported as percentage of the methylated (C) allele over the background of un-methylated (T) allele. All primers and probes for HGF and c-Met are listed in Table S2.

### Statistical analysis

Mouse experiments involved a total of 10 mice per cohort as follows: Subcutaneous implantation experiments involved 7 mice per cohort. Survival surgical intrahepatic implantation experiments involved 3 mice per cohort. In additional separate experiments, 5 mice each were used to compare the tumorigenicity of BNL 1ME A.7R.1 cells, OL2548 cells and OL2549 cells. qPCR (normalized to GAPDH) experiments were performed in triplicates. Results are expressed as mean ± SEM. Effects were compared with controls. Paired *t* tests were used to analyze the effect of experiments in comparison to controls. *P*<0.05 was considered significant.

## Results

### Establishment of novel circulating tumor cell (CTC) lines

**Figure 1:**
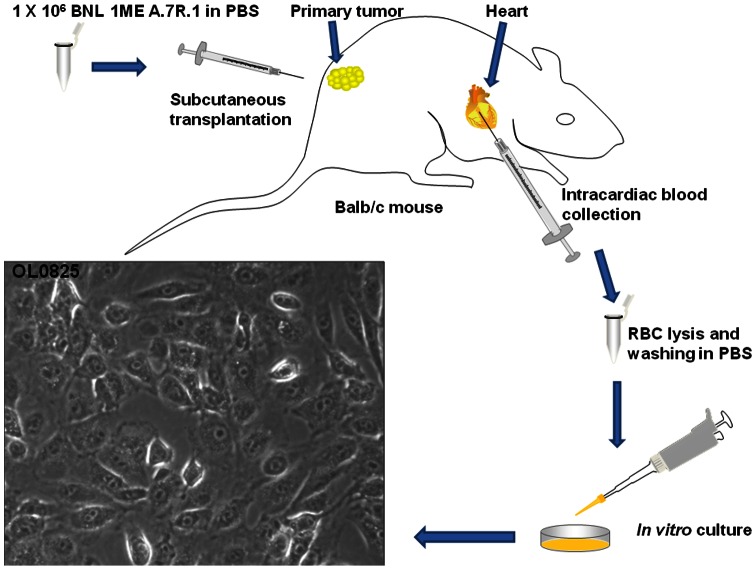
Summary of novel methodology for establishment of OL0825 CTC line. Subcutaneous implantation of 1×10^6^ BNL 1ME A.7R.1 cells into the flank of a Balb/c mouse resulted in tumor development in the flank. Humane euthanization of mouse involved CO_2_ asphyxiation and intracardiac exsanguination. 500–1000 µl of whole blood was obtained. After centrifugation, buffy coat layer was subjected to red blood cell lysis, washed in PBS and cultured in medium in a humidified incubator at 37°C with air and 5% CO_2_. Tumor cell proliferation was observed within 48 hours. The cell line was designated OL0825 and is still very viable after 25 passages and repeated freezing and thawing. Phase contrast image of OL0825 was taken at ×40 magnification (objective lens).

In order to determine if we can isolate and culture CTCs from blood, we used a syngeneic mouse model of HCC. One million BNL 1ME A.7R.1 cells (HCC cell line from Balb/c mouse) were subcutaneously implanted into Balb/c mice. This model is well established in our laboratory [20], [26]. Further, we developed a novel syngeneic model involving survival surgical implantation of BNL 1ME A.7R.1 cells into liver of Balb/c mice. After tumor development, 500–1000 μl of whole blood from the mice was collected and processed as detailed in the methods section. Isolated cells were washed with PBS and seeded into tissue culture dishes. After 48 hours of culture, we observed that some cells had adhered to the dish and were proliferating. These cells remain viable and continue to proliferate even after 25 passages as well as after repeated freezing and thawing. We have now successfully established 3 novel HCC CTC lines: OL0825 (Figure 1) and OL2549 and OL2548 (Figure S1). A novel PCR-based method that amplifies only one specific DNA segment of the mouse β-globin gene clearly verified that the novel established circulating tumor cell lines (OL0825, OL2548 and OL2549) are mouse cells just like the originally implanted BNL 1ME A.7R.1 HCC line. Neither the Huh7 human HCC cell line nor distilled water showed any amplification, thus confirming specificity and accuracy of the assay (Figure S2). Furthermore, immunostaining for the hepatocyte-specific marker CREB3L3 using a specific antibody revealed that BNL 1ME A.7R.1, OL0825, OL2548 and OL2549 cells all express CREB3L3 thus verifying that the CTC lines are from the originally implanted BNL 1ME A.7R.1 cell line (Figure S3).

### Circulating tumor cells have increased tumorigenicity and metastatic potential

**Figure 2:**
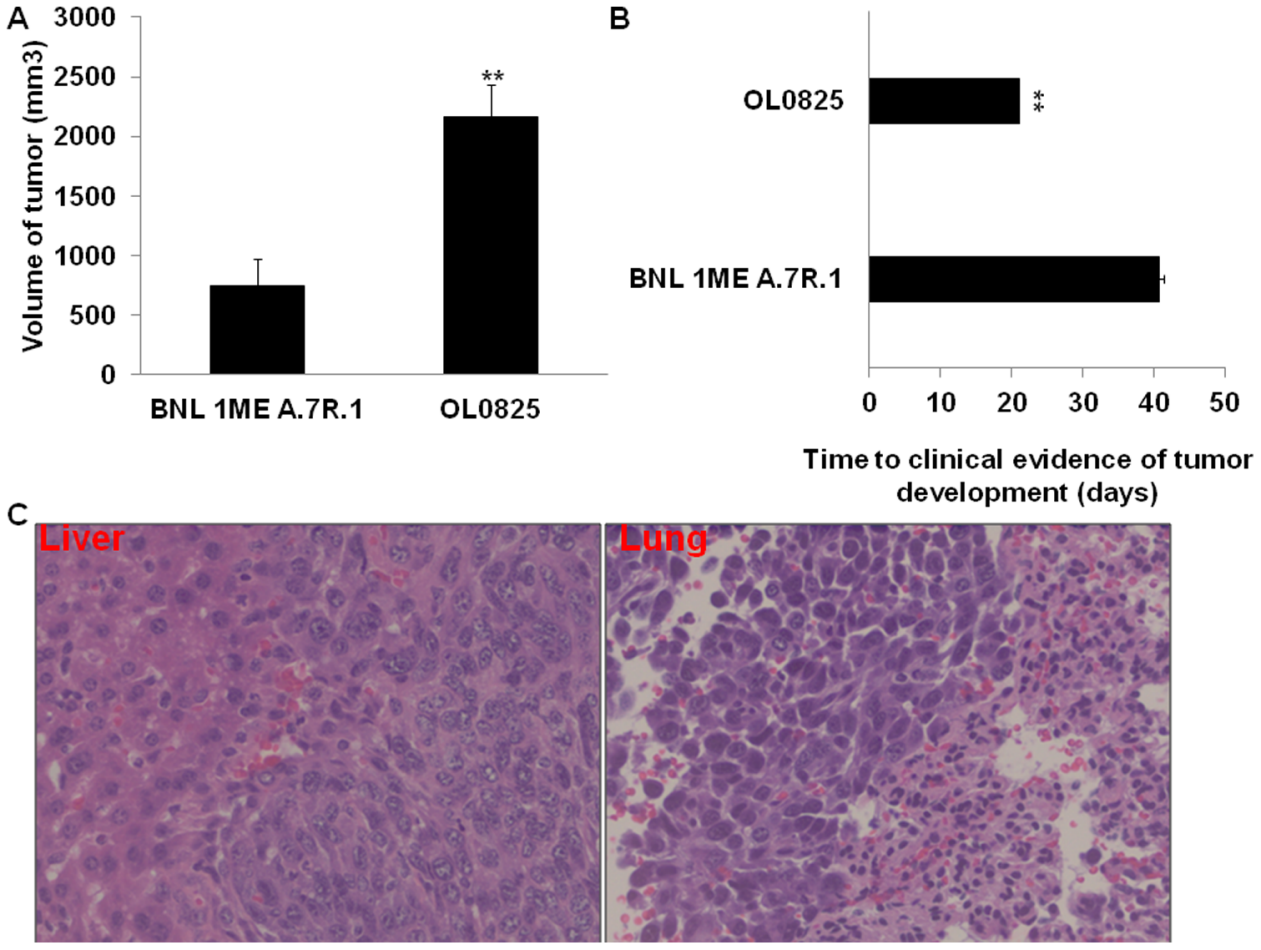
Circulating tumor cells have increased tumorigenicity. 1×10^6^ BNL 1ME A.7R.1 or OL0825 cells were subcutaneously implanted into 7 separate Balb/c mice or surgically implanted into the liver of 3 separate Balb/c mice. **A**. Tumors from OL0825 had significantly greater volume than those from BNL 1ME A.7R.1. **B**. OL0825 cells resulted in tumor development twice as fast as BNL 1ME A.7R.1 cells in the survival surgical intrahepatic implantation model. **C.** Representative images of orthotopic tumor in the liver and metastatic lesions in the lung from implantation of OL0825 into liver of immune competent wild type Balb/c mice. Images of H&E-stained slides were acquired at ×40 (objective lens). Results presented as mean ± SEM; P<0.01.

We performed separate subcutaneous and survival surgical hepatic implantation of 1×10^6^ BNL 1ME A.7R.1 or OL0825 cells into separate Balb/c mice. Subcutaneous implantation of OL0825 cells resulted in tumors with greater volume (3-fold) than tumors from subcutaneous implantation of BNL 1ME A.7R.1 cells (Figure 2A). Also, in additional subcutaneous implantation experiments comparing BNL 1ME A.7R.1 cells with OL2548 and OL2549 cells, we found that tumors from OL2548 (1.4-fold) and OL2549 (2.8-fold) cells have greater volume than tumors from BNL 1ME A.7R.1 cells (Figure S1). Further, analysis of tumor development from survival surgical hepatic implantation of BNL 1ME A.7R.1 and OL0825 cells revealed that mice implanted with OL0825 demonstrated clinical evidence of tumor development twice as fast as mice implanted with BNL 1ME A.7R.1 (Figure 2B). Importantly, orthotopic implantation of OL0825 (detected at 3 weeks) and BNL 1ME A.7R.1 (detected at 6 weeks) cells into the liver of immune competent wild type Balb/c mice resulted in the development of metastatic tumors to the lungs in 4 out of 6 cases (see Figure 2C). In a separate experiment, we isolated blood-derived CTCs from a Balb/c mouse that developed a tumor from BNL 1ME A.7R.1 implantation. Then, we immediately subcutaneously implanted them directly into another Balb/c mouse without any *in vitro* culture. Interestingly, we observed that the mouse developed a tumor, abdominal distension, became thin, hunched, dehydrated and pale. Consequently, we sacrificed it and examined the tumor, liver and lungs. We found macroscopic evidence of metastasis in the liver and lungs as shown in figure S4. Subcutaneous implantation of BNL 1ME A.7R.1 has never been reported to result in metastasis. Hematoxylin and eosin (H&E) staining analysis of tissues from the primary tumor, liver and lungs of the Balb/c mouse subcutaneously implanted with the blood-derived CTCs confirmed the dissemination of metastatic HCC lesions to the liver and lungs of the mouse (Figure S4).

### Circulating tumor cells have increased mesenchymal characteristics

**Figure 3:**
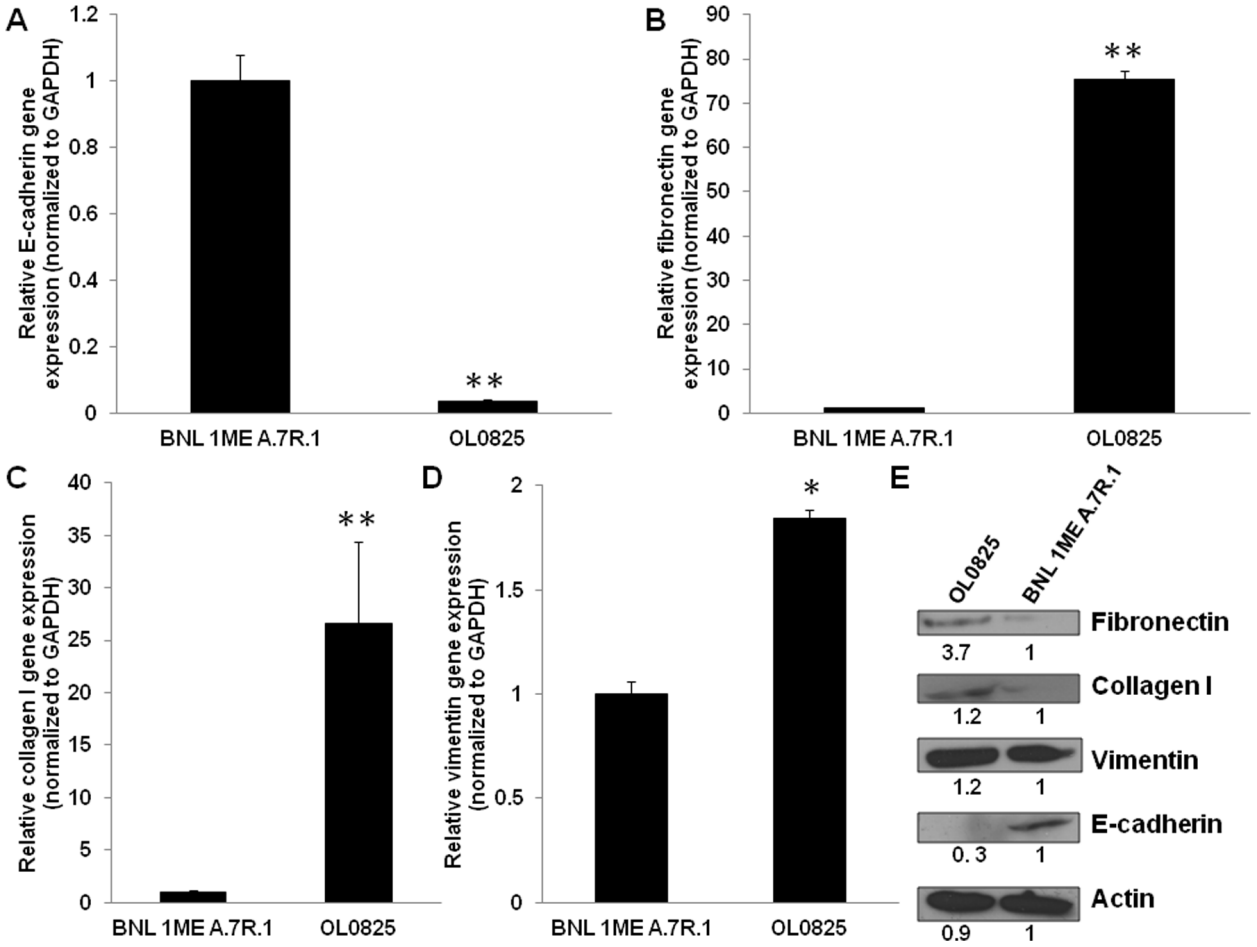
Circulating tumor cells have increased mesenchymal characteristics. Analysis of gene and protein expression of markers of epithelial-mesenchymal transition were performed using qPCR and Western blotting respectively. There is significantly decreased E-cadherin gene expression (**A**), increased fibronectin gene expression (**B**), increased collagen I gene expression (**C**) and increased vimentin gene expression (**D**) by OL0825 than BNL 1ME A.7R.1 cells. **E**. OL0825 demonstrate increased fibronectin, collagen I, vimentin and decreased E-cadherin protein expression in comparison to BNL 1ME A.7R.1 cells. Relative densitometry is indicated below the bands. Results presented as mean ± SEM; P<0.05; P<0.01; N = 3.

We also wanted to determine if epithelial-mesenchymal transition (EMT) plays a role in hematogenous dissemination of tumor cells. To do this we performed a comparative analysis of the expression of markers of EMT in BNL 1ME A.7R.1 cells versus OL0825 cells. The expression pattern of E-cadherin, fibronectin, collagen I and vimentin were analyzed using real-time quantitative reverse transcription polymerase chain reaction (qPCR) and Western blotting. Our analyses revealed that the blood-derived CTC line OL0825 has significantly increased mesenchymal characteristics in comparison to BNL 1ME A.7R.1 cells. This is clearly shown in figure 3 by decreased E-cadherin gene and protein expression and increased fibronectin, collagen I and vimentin gene and protein expression.

### Circulating tumor cells have increased HGF and c-Met expression

**Figure 4:**
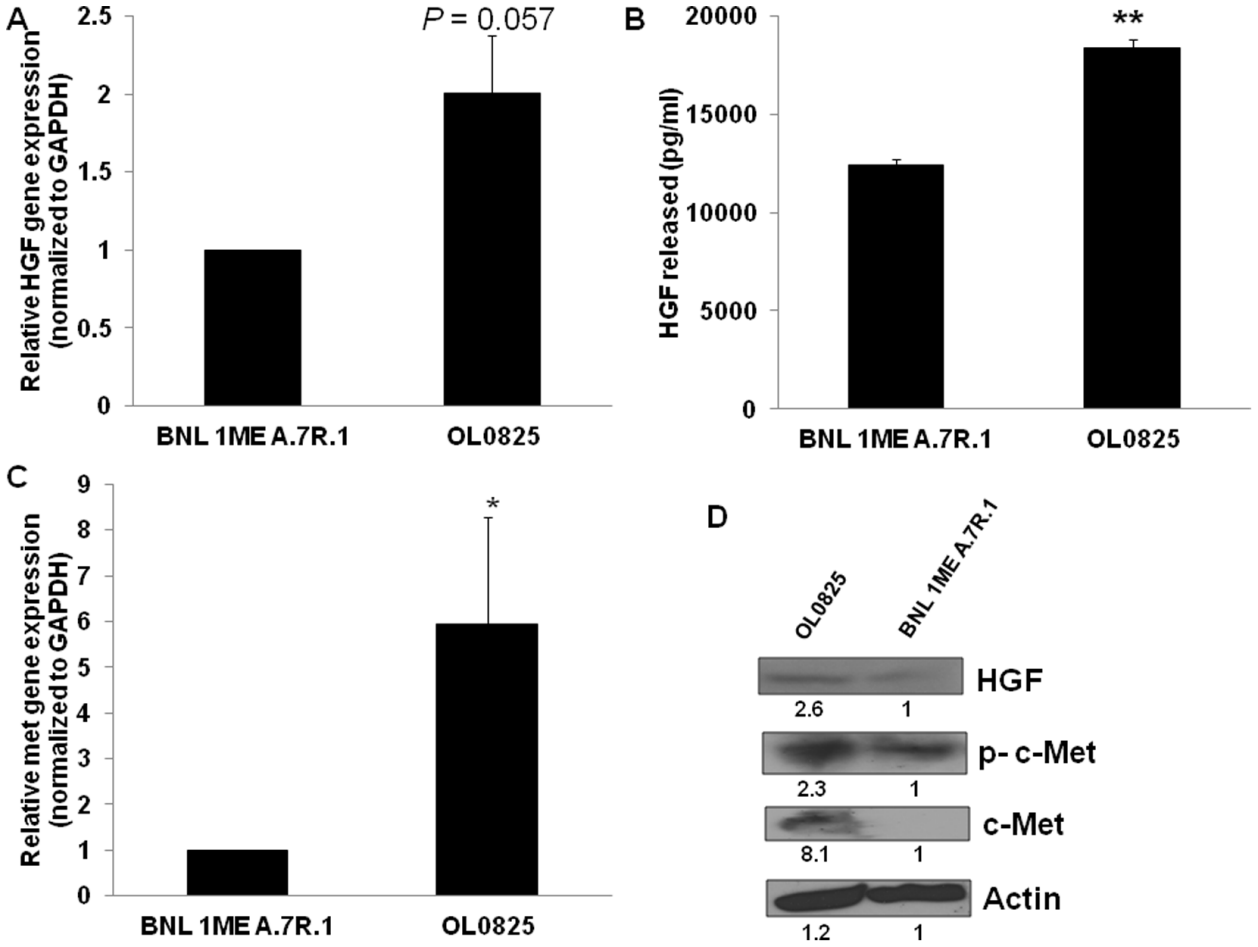
HGF and c-Met overexpression in HCC CTCs. Analysis of gene expression of HGF and c-Met was performed by qPCR. c-Met protein expression analysis was performed by Western blotting. Analysis of HGF protein expression and secretion were performed by Western blotting and ELISA respectively. Gene expression of HGF (**A**) and c-Met (**C**) is increased in OL0825 in comparison to BNL 1ME A.7R.1. **B**. HGF protein secretion is significantly higher in OL0825 cells than in BNL 1ME A.7R.1 cells. **D**. OL0825 has increased protein expression of HGF, c-Met and phosphorylated c-Met (p-c-Met) in comparison to BNL 1ME A.7R.1. Relative densitometry is indicated below the bands. Results presented as mean ± SEM; P<0.05; P<0.01; N = 3 (for qPCR); N = 6 (for HGF ELISA).

Recent studies suggest that hepatocyte growth factor (HGF) up regulation is associated with acquisition of mesenchymal characteristics, development of HCC and metastasis [20], [27]–[29]. However, the role of HGF and its receptor the proto-oncogene c-Met in hematogenous dissemination of HCC has never been studied. Consequently, we determined if the viability of CTCs and their increased mesenchymal characteristics are related to HGF and c-Met. We compared the gene and protein expression of HGF and c-Met and found that HGF and c-Met expression in the novel blood-derived CTC line OL0825 were significantly higher than in the parent BNL 1ME A.7R.1 cell line. Notably, HGF released into medium and phosphorylation of c-Met were also higher in OL0825 than BNL 1ME A.7R.1. These data are shown in figure 4.

### HGF induces EMT in BNL 1ME A.7R.1 cells

**Figure 5:**
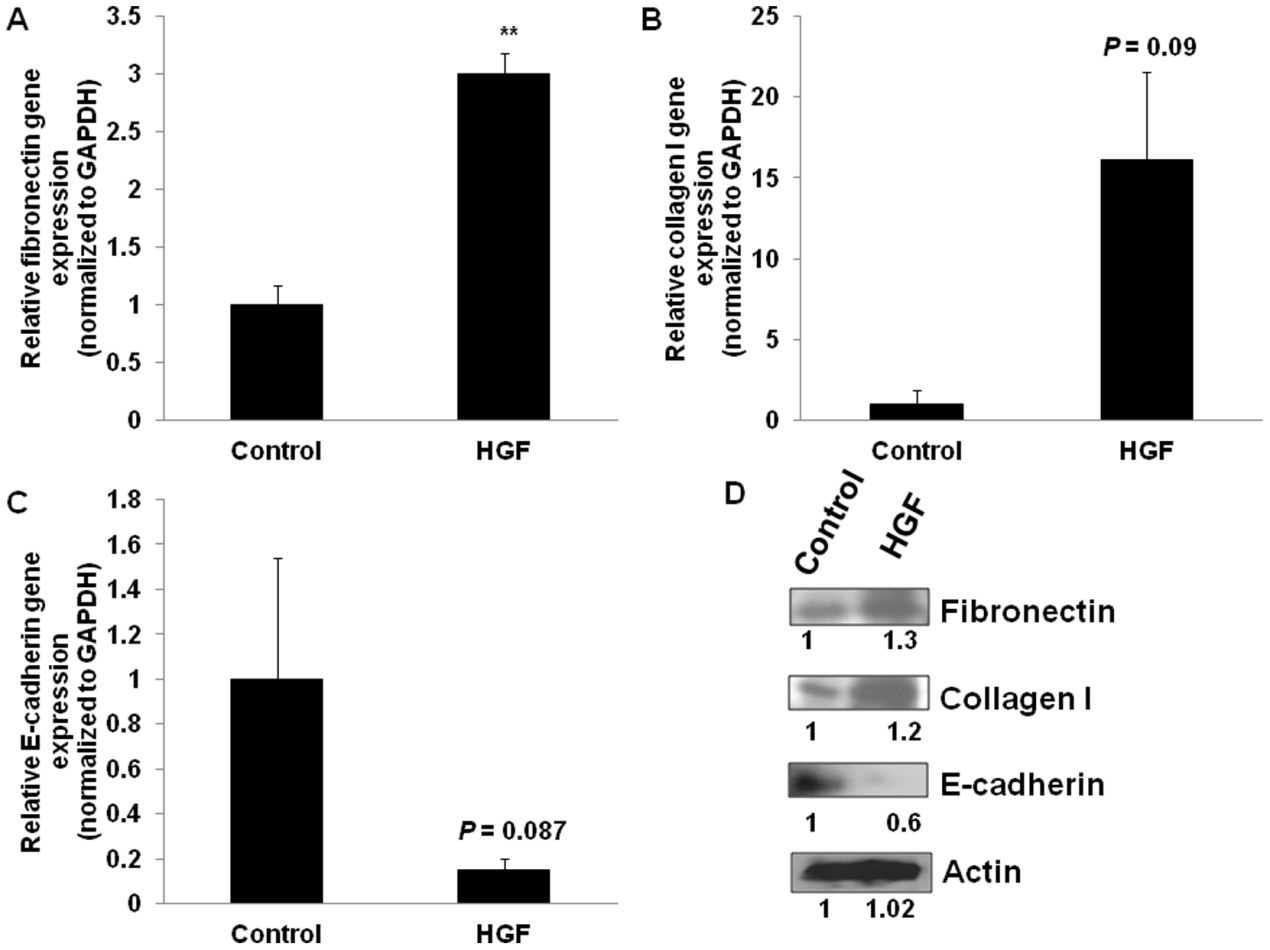
HGF induces EMT in BNL 1ME A.7R.1 cells. BNL 1ME A.7R.1 cells were cultured in 6-well plates and treated with 25 ng/ml mouse HGF (R & D Systems) for 5 days. Control cells were untreated. RNA and protein extraction were performed and qPCR and Western blotting were performed as described in “Experimental procedures” section. HGF-treated BNL 1ME A.7R.1 cells demonstrated increased fibronectin gene and protein expression (**A**, **D**), increased collagen I gene and protein expression (**B**, **D**) and loss of E-cadherin gene and protein expression (**C**, **D**). Relative densitometry is indicated below the bands (**D**). Results presented as mean ± SEM; P<0.01; N = 3.

To determine if the increased mesenchymal characteristics observed in the OL0825 CTC line is causally related to the overexpression and increased secretion of HGF observed, we analyzed the effects of directly exposing BNL 1ME A.7R.1 cells to HGF on expression of molecular markers of EMT. Treatment of 1MEA cells with HGF resulted in increased gene expression of fibronectin (about 3-fold), collagen I (about 15-fold) and decreased expression of E-cadherin (about 5-fold) (Figure 5). These data were confirmed by analysis of protein expression with Western blotting.

### HGF and c-Met promoter demethylation in circulating tumor cells

**Figure 6:**
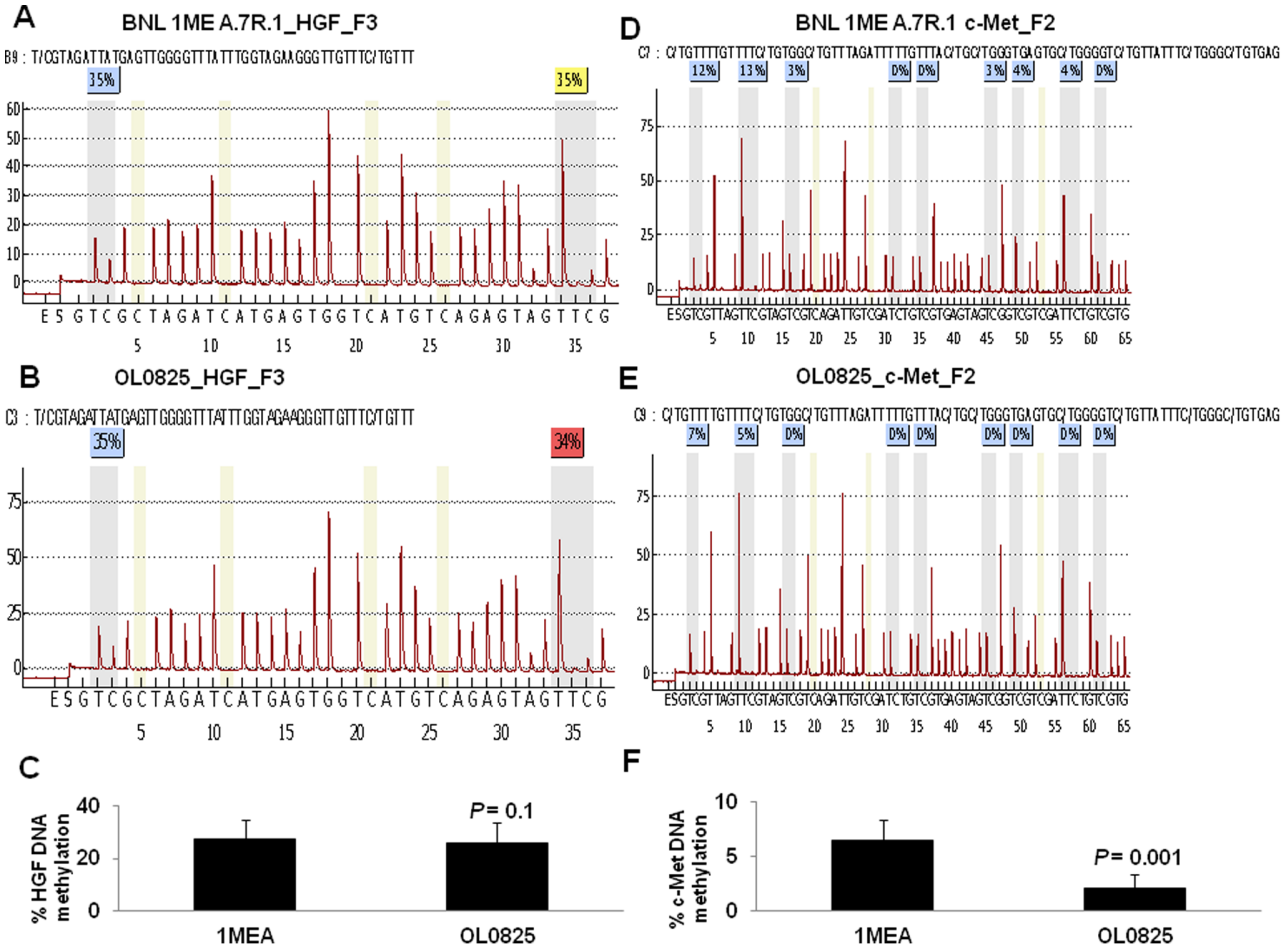
DNA methylation analysis of HGF and c-Met promoters. **A**. Analysis of bisulfite DNA from BNL 1ME A.7R.1 cells indicates that HGF has 35% of CpGs methylated at two sites. **B**. Analysis of bisulfite DNA from OL0825 CTC line shows that the HGF has methylation percentages of 35% and 34% detected at the first and second sites respectively. **C**. Statistical analysis of the percentage of CpGs methylated in the HGF amplicon (including data from a third CpG site analyzed in Figure S6) shows that there is a small difference between the methylation of BNL 1ME A.7R.1 and OL0825 cells. **D**. Methylation analysis of c-Met from BNL 1ME A.7R.1 cells indicates the presence of methylation in 6 CpGs including the first 2 CpGs (which are close to 2 putative transcriptional start sites). The percentages of methylation at these first 2 sites are 12% and 13% respectively. **E**. Methylation analysis of c-Met in OL0825 cells indicates that c-Met has lower methylation than that of BNL 1ME A.7R.1 cells, with the first 2 CpGs being methylated at only 7% and 5% respectively. **F**. Statistical analysis of the differences between methylation levels in BNL 1ME A.7R.1 and OL0825 cells shows that the data are highly significant. Results presented as mean ± SEM; N = 3 for HGF and N = 6 for c-Met.

To further examine the molecular mechanism by which HGF and c-Met are regulated, we performed DNA methylation analysis using pyrosequencing approach. Two primer pairs were designed, one pair for the c-Met promoter and one pair for the HGF promoter; c-Met_F2 and HGF_F3 respectively. These primers were optimized as part of a comprehensive primer set (Table S2) designed to tile over the promoters of HGF and c-Met to analyze DNA methylation by high resolution melt (HRM) analysis using commercial DNA samples as controls. OL0825 cells have decreased level of HGF DNA methylation in comparison to BNL 1ME A.7R.1 cells as determined by HRM analysis (Figures S6 and S7). Pyrosequencing analysis (Figure 6 and Figures S8 and S9), however, revealed only a minor loss of DNA methylation at 2 of 3 CpG sites analyzed thus suggesting that loss of DNA methylation alone is likely not solely responsible for the increase in HGF expression in the CTC line OL0825. However, DNA methylation at the c-Met promoter is significantly lower in OL0825 cells when compared to BNL 1ME A.7R.1 cells as determined by both HRM analysis (Figures S6 and S7) and pyrosequencing (Figure 6 and Figure S8). This definitively indicates that a loss of DNA methylation is associated with the increased expression of c-Met in the CTC line OL0825: differences were observed at 6 of the 9 CpGs analyzed (see Figure 6 and Figure S10).

## Discussion

Metastasis is the most important cause of mortality due to cancer. Distant metastatic spread of solid cancers is mostly due to hematogenous dissemination. The mechanisms of hematogenous dissemination are, however, unclear. To our knowledge, there have been no previous reports of any syngeneic whole animal experimental model that can be used to reproducibly analyze the regulatory mechanisms of circulating tumor cells in cancer metastasis. Despite current efforts to understand the biology of tumors at the primary site and at secondary sites, cancer is still the second largest killer in the US. Consequently, it is likely that a novel approach involving understanding the biology of circulating tumor cells (CTCs) and the mechanisms of hematogenous dissemination may reveal novel insights into the mechanisms of cancer metastasis.

Here, for the first time, we report the role and regulatory mechanisms of HGF and its receptor, c-Met, in CTC biology during hematogenous metastasis of HCC. This study was possible because of successful establishment of novel CTC lines from a syngeneic murine model of HCC. To our knowledge, these are the very first CTC lines from a syngeneic solid cancer model. They are, therefore, novel tools that can be used to probe the biology of CTCs and their role in metastatic spread of solid cancers. Importantly, the method described here for the isolation and culture of CTCs has potential for clinical application as a non-invasive approach for early detection, prognostication, monitoring and personalizing therapy in metastatic solid cancers.

Cell-based and animal studies suggest that epithelial-mesenchymal transition (EMT) plays an important role in tumor progression and metastasis. This has, however, been questioned by a few studies that suggest the possibility of cancer progression independent of EMT. For example, Pinkas and Leder studied a model of mammary tumorigenesis wherein activation of mitogen activated protein kinase 1/2 (MAPK 1/2) in an epithelial cell line resulted in metastatic tumors [5]. Re-isolation and analysis of the tumorigenic cells revealed that constitutive activation of MAPK 1/2 was maintained as well as epithelial cell characteristics. It was, however, unclear whether the cells from the metastatic sites were analyzed as to whether they had acquired mesenchymal characteristics. Importantly, neither that study nor others since have definitively studied and determined the status of CTCs with respect to the role of EMT in tumor progression and metastasis. Consequently, the present study specifically determined whether the novel CTC line OL0825 has acquired mesenchymal characteristics in comparison to the original implanted primary tumor cell line, BNL 1ME A.7R.1. The data clearly show loss of E-cadherin expression associated with gain of fibronectin, collagen I and vimentin expression in OL0825 cells in comparison to BNL 1ME A.7R.1 cells. Thus, OL0825 cells have increased mesenchymal characteristics in comparison to BNL 1ME A.7R.1. This suggests that intravasation of tumor cells into blood circulation and continuing viability during hematogenous circulation may be associated with tumor cells undergoing EMT.

We also focused on determining the specific molecular factors that may be driving this transition to a more mesenchymal phenotype in CTCs. In a previous study comparing the BNL 1ME A.7R.1 cell line with the BNL.CL.2 non-tumorigenic hepatocyte cell line from which it was derived, we had observed 200-fold increased gene expression and almost 20-fold increased protein secretion of HGF [20]. Moreover, many other studies have implicated HGF and its receptor, the c-Met proto-oncogene, in tumor progression and metastasis in HCC [27], [30]–[32]. HGF/c-Met has also been implicated in the progression and metastasis of other solid cancers [33]–[35]. The involvement of HGF/c-Met signaling in hematogenous dissemination of cancer cells, however, has never been previously reported. Consequently, we hypothesized that HGF and c-Met may play a role as drivers of EMT in CTCs. We tested this hypothesis by analyzing the expression pattern of HGF and c-Met in both BNL 1ME A.7R.1 and OL0825 cells. We found increased HGF and c-Met expression at both gene and protein levels and significantly increased HGF release by OL0825 cells in comparison to BNL 1ME A.7R.1 cells. Thus, the data suggest that increased HGF and c-Met expression may be important factors promoting EMT-associated hematogenous dissemination in HCC. Further, the increased HGF release by the OL0825 CTC line suggests that the mechanism may involve autocrine and paracrine binding of HGF to elevated c-Met on CTCs thus triggering downstream signaling. This explanation is further strengthened by the fact that we observed that HGF treatment does indeed induce EMT in BNL 1ME A.7R.1 cells. It is noteworthy that c-Met overexpression in this model appears to be specific to CTCs. In our previous study of c-Met expression between the BNL 1ME A.7R.1 cell line and the BNL.CL.2 non-tumorigenic hepatocyte cell line from which it was directly established, we observed that there was no significant difference in c-Met expression between the two cell lines [20]. This analysis has since been repeated and the findings remain consistent. Thus, it is likely that c-Met overexpression in tumor cells is a specific factor promoting hematogenous dissemination and metastasis in HCC. The role of c-Met in the progression and metastasis of many cancers has been described and there are now a number of c-Met inhibitors in clinical trials [28]–[33], [35]–[38]. It is, therefore, possible that in the near future more effective treatment of cancer patients may involve non-invasive isolation and analysis of individual patient CTCs as described here. Further, personalized treatment for HCC patients with c-Met overexpression positive CTCs can be offered using a c-Met inhibitor.

Cancer metastasis via hematogenous dissemination of tumor cells is believed to be an inefficient process with the vast majority of cells unable to survive and only a small subpopulation remaining viable and capable of initiating tumors at secondary sites. The inefficiency of the process is likely partly due to the immune surveillance actions of T lymphocytes and other components of the immune system in blood. It is, therefore, likely that significant epigenetic dysregulation may be associated with and may even be required for the viability of CTCs and their ability to ultimately initiate tumors at secondary sites. Consequently, we hypothesized that the significant overexpression of c-Met and HGF release observed may be due to dysregulation of promoter methylation. Initial analysis of BNL 1ME A.7R.1 cells treated with 5-aza-2′-deoxycytidine (AZA) revealed that AZA treatment resulted in increased protein expression of HGF and c-Met (see Figure S5), thus indicating that the increased expression observed in OL0825 may be DNA methylation-dependent. Consequently, we proceeded to analyze the role of promoter methylation in the dysregulation of HGF and c-Met in a rigorous fashion. High resolution melt analysis with the most optimal primers revealed promoter demethylation in both HGF and c-Met in OL0825 cells in comparison to BNL 1ME A.7R.1 cells. Although pyrosequencing showed only a minor decrease in HGF DNA methylation at 2 of the 3 CpG sites in OL0825 cells, the decrease in c-Met DNA methylation was widespread and profound: Decreased DNA methylation was observed at 6 of the 9 CpG sites. This indicates that whereas DNA methylation status is likely not the only mechanism regulating HGF overexpression in OL0825 cells, c-Met overexpression is definitely associated with DNA promoter demethylation. This may be very important clinically because dysregulation of DNA methylation has been implicated in the progression of solid cancers including HCC [39]–[41]. Moreover, there is evidence that DNA methylation analysis is applicable clinically for screening, diagnosis and prognostication in cancer [42]. Furthermore, the fact that all the 6 CpG sites implicated in c-Met overexpression are in the promoter region of the gene and the first 2 CpG sites analyzed (−566 and −555) are included among the 6 is very significant indeed. This novel mechanistic insight into the regulation of c-Met in HCC may present a novel opportunity for non-invasive clinical applications as there was previously only limited information on the role of DNA methylation in regulating c-Met in cancer.

Although previous studies suggest that c-Met is regulated by a number of transcription factors including p53 [43], there is previously very little information on the role of DNA methylation in regulating c-Met. In 2008, Morzov and colleagues demonstrated that c-Met was also in part regulated by Daxx, a transcriptional repressor binding primarily to the first exon and promoter activation region of the gene, and showed evidence that Daxx binding caused repression by H4 acetylation on the c-Met promoter in murine fibroblasts [44]. They also performed DNA methylation analysis of c-Met by traditional cloning and sequencing but they did not find evidence for regulation by DNA methylation in their model of Daxx ^−/−^ and Daxx ^+/+^ cells [44].

In our study we have used high resolution melt analysis and pyrosequencing to analyze the DNA methylation of the c-Met promoter to clearly determine the role of DNA methylation on expression of c-Met. These methods are not prone to biases which can arise from bisulfite-specifc PCR and cloning which favor amplification of unmethylated DNA [45]. Expression of c-Met appears to be directly linked to the level of DNA methylation on the promoter of c-Met in BNL 1ME A.7R.1 cell line and the OL0825 CTC line. There are two ATG transcription initiation sites located at −597 and −571 to the transcription start site. The two CpGs closest to the initiation codons (−566 and −555) appear to have the biggest effect on transcription at the c-Met promoter. This signifies the importance of DNA methylation at these 2 CpGs, which alters the local transcriptional environment and may explain why OL0825 cells having less methylation at these sites results in an increased expression of c-Met relative to BNL 1ME A.7R.1 cells.

**Figure 7:**
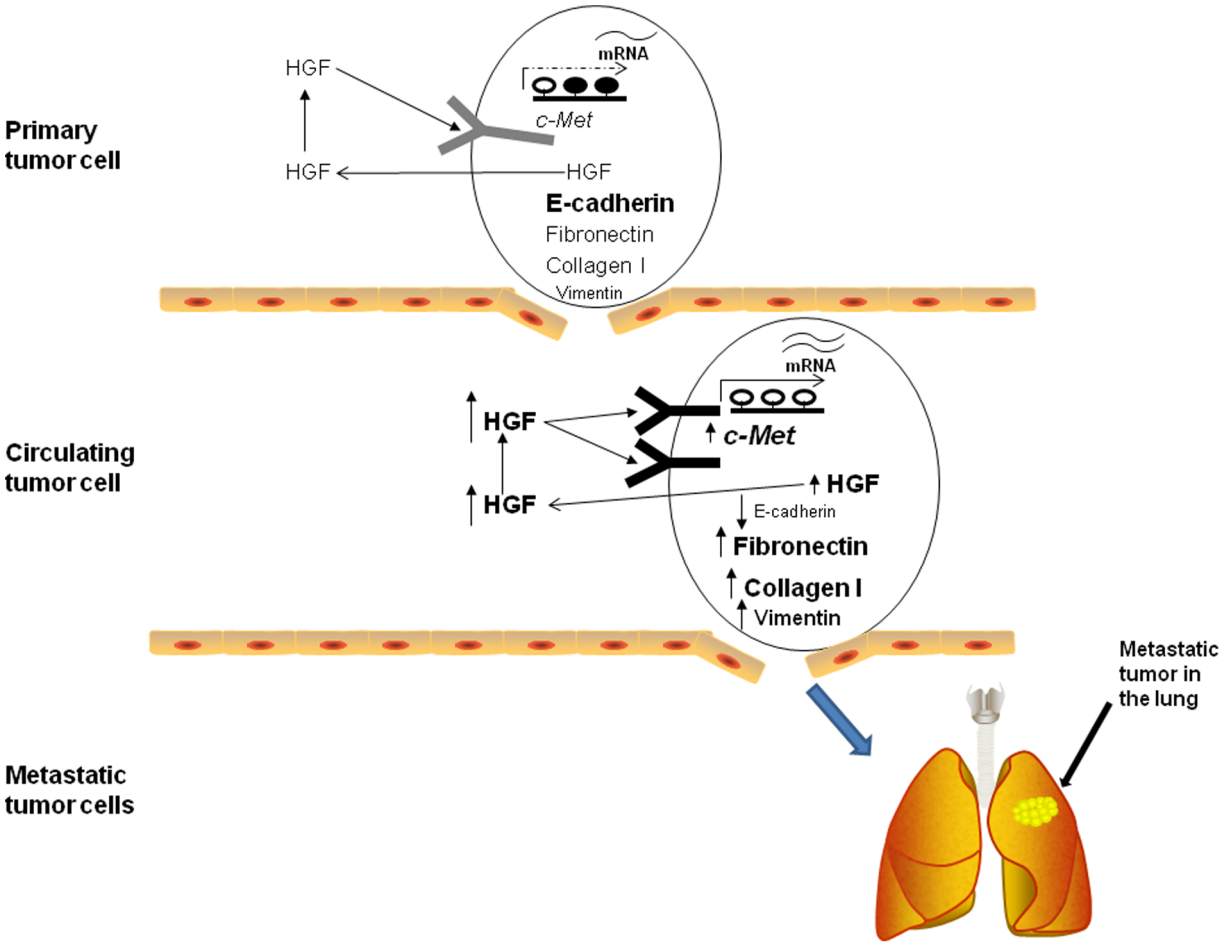
Summary of potential role of HGF/c-Met in hematogenous dissemination of HCC. Schematic diagram illustrating the role of HGF/c-Met in hematogenous dissemination of HCC cells. Primary tumor cells express HGF and c-Met with partial promoter demethylation. This is associated with good expression of E-cadherin and limited expression of fibronectin, collagen I and vimentin. However, when HCC cells escape from the liver and start circulating in the blood-stream, increased expression of HGF and c-Met associated with increased promoter demethylation is observed. Circulating HCC cells also display evidence of EMT: loss of E-cadherin, increased fibronectin, increased collagen I and increased vimentin expression. We conclude that increased HGF and c-Met may induce EMT in CTCs to sustain hematogenous dissemination.

*In vitro* viability of our novel CTC line was confirmed by sustained viability after over 25 passages and repeated freezing and thawing. We then investigated *in vivo* viability and functional significance of the novel cell line by subcutaneous and survival surgical intrahepatic implantation into immune competent mice. The data not only show that the novel CTC line OL0825 is viable *in vivo*, but more importantly OL0825 cells are more tumorigenic and metastatic than BNL 1ME A.7R.1 cells. Tumors developed had significantly increased volume and time to tumor development was reduced by 50%. Thus, we conclude that DNA promoter demethylation-dependent upregulation of HGF and c-Met observed in OL0825 CTCs drives the increased tumorigenicity and metastatic capacity of the OL0825 CTC line (summarized in figure 7). Further, we directly implanted isolated CTCs that had not been cultured *in vitro* into an immune competent mouse. We observed that a primary tumor developed at site of subcutaneous implantation and macroscopic tumors were visible in the liver and lungs and these were confirmed by histology. Implantation of BNL 1ME A.7R.1 cells into wild type immune competent Balb/c mice has never previously resulted in metastatic tumors. This is, therefore, the first report of both orthotopic and subcutaneous syngeneic metastatic HCC in the wild type immune competent Balb/c mouse. Importantly, our data shows that CTCs in these syngeneic murine models definitely possess enhanced tumor initiating and metastatic capacity. Moreover, it demonstrates the relevance of these syngeneic models for the study of the role of hematogenous dissemination in metastatic spread of solid cancers. It will, therefore, prove to be a useful platform for understanding the biological mechanisms underlying hematogenous dissemination and metastasis in HCC and potentially for developing study of CTCs for non-invasive clinical applications.

It is noteworthy that isolated CTCs are likely heterogeneous and may remain so even when they become established cell lines. Consequently, the inclusion of multiple subclones and passages of the novel CTC lines in future studies is likely to illuminate this field further and is encouraged.

Nevertheless, this approach to studying cancer metastasis is very promising. The findings from the current study provide strong basis for promoting the utilization of this novel approach for studying the mechanisms of metastasis of solid cancers.

## Supporting Information

Figure S1 **OL2549 and OL2548 are novel CTC lines. Top.** One million BNL 1ME A.7R.1 cells were implanted directly into the liver of separate Balb/c mice via survival surgery as described in the “materials and methods” section. The Balb/c mice were humanely euthanized when clinical evidence of tumor development was observed. Isolation of CTCs was performed as described in the “materials and methods” section. Novel CTC lines designated OL2549 and OL2548 were established. They are still viable after multiple passages. Phase contrast images at ×40 magnification (objective lens). **Bottom.** 1×10^6^ BNL 1ME A.7R.1 or OL2548 or OL2549 cells were subcutaneously implanted into 5 separate Balb/c mice. Tumors from OL2548 and OL2549 cells had greater volume than those from BNL 1ME A.7R.1 cells.
(TIF)

Figure S2 **CTC lines and BNL 1ME A.7R.1 cells are all of mouse origin**. Image of ethidium bromide-stained 1.2% agarose gel showing mouse-specific expression of a specific segment of the DNA of the mouse β-globin gene. Specificity is confirmed by lack of expression by the Huh7 human HCC cell line and by the distilled water negative control. The DNA marker used was the 100bp.
(TIF)

Figure S3 **CTC lines and BNL 1ME A.7R.1 cells all express specific marker of hepatocytes**. Images of BNL 1ME A.7R.1, OL0825, OL2548 and OL2549 cells specifically stained for expression of the hepatocyte-specific marker CREB3L3. Image magnification was ×20 objective. Green coloration represents CREB3L3 expression and blue coloration represents DAPI-stained nuclei.
(TIF)

Figure S4 **Circulating tumor cells have increased metastatic potential.** CTCs were isolated from a Balb/c mouse that developed tumor from implantation of BNL 1ME A.7R.1 cells using the method described in the “materials and methods” section. Isolated CTCs were washed in PBS, suspended in PBS and implanted subcutaneously into the left flank of another Balb/c mouse. Tumor developed in the left flank at site of implantation (**F**). Macrometastasis was observed in the lungs (**D**) and liver (**E**). Hematoxylin and eosin staining of formalin-fixed paraffin-embedded sections revealed histologic evidence of primary tumor at left flank mass (**A**), metastatic tumor in liver (**B**) and lungs (**C**). Bright field images were taken at ×40 magnification (objective lens).
(TIF)

Figure S5 **Treatment of BNL 1ME A.7R.1 cells with 5-aza-2**′**-deoxycytidine (AZA) results in increased expression of HGF and c-Met.** BNL 1ME A.7R.1 cells were cultured in complete culture medium as described in the “materials and methods” section. At 60–70% confluency, cells were treated with 5 µM AZA for 24 hours. Protein expression of phosphorylated c-Met (p-c-Met), c-Met, and HGF were analyzed with Western blotting. Relative densitometry is indicated below the bands.
(TIF)

Figure S6 **High resolution melt analysis of HGF and c-Met promoters.** Melt analysis of the murine HGF promoter and mouse c-Met promoter using HGF_F3 and c-Met_F2 primers indicates that there is a temperature difference between the PCR products from BNL 1ME A.7R.1 cells and OL0825 cells. HGF products from BNL 1ME A.7R.1 cells are 76.5°C (**A**) whereas products from OL0825 cells have a melt temperature 76.21°C (**B**) which indicates that OL0825 cells have less methylation than BNL 1ME A.7R.1 cells. c-Met products from BNL 1ME A.7R.1 cells are 77.55°C (**C**) and products from OL0825 cells are 77.25°C (**D**) indicating that OL0825 cells have less methylation than 1MEA cells.
(TIF)

Figure S7 **Quantitative analysis of HRM analysis of HGF and c-Met promoters.** The individual melt temperatures of the PCR replicates were plotted on a bar chart. The data shows decreased melt temperatures for both HGF(**Left**) and c–Met (**Right**) in OL0825 cells in comparison to BNL 1ME A.7R.1 cells. Results presented as mean ± SEM; P<0.05; N = 3.
(TIF)

Figure S8 **Pyrosequencing analysis of HGF DNA promoter region at first CpG site.** The data shows only 2% reduction in DNA methylation at this CpG site between BNL 1ME A.7R.1 and OL0825 cells.
(TIF)

Figure S9 **Pyrosequencing analysis of HGF DNA promoter region at two other CpG sites.** The data shows only 1% reduction in DNA methylation at one of the two CpG sites between BNL 1ME A.7R.1 and OL0825 cells.
(TIF)

Figure S10 **Pyrosequencing analysis of c-Met promoter region (9 CpG sites).** The data shows clear reduction in DNA methylation at 6 of 9 CpG sites between BNL 1ME A.7R.1 and OL0825 cells.
(TIF)

Table S1 **Primers for a DNA segment of the mouse β-globin gene.**
(DOCX)

Table S2 **Primers and probes for pyrosequencing.**
(DOCX)

Table S3 **Primers and probes for c-Met and HGF promoter analysis.**
(DOCX)
