# Supplementary figures and images for: Epigenetic Upregulation of HGF and c-Met Drives Metastasis in Hepatocellular Carcinoma
Source: PLoS One. 2013 May 28;8(5):e63765. doi: 10.1371/journal.pone.0063765 (PMC3665785; doi:10.1371/journal.pone.0063765)

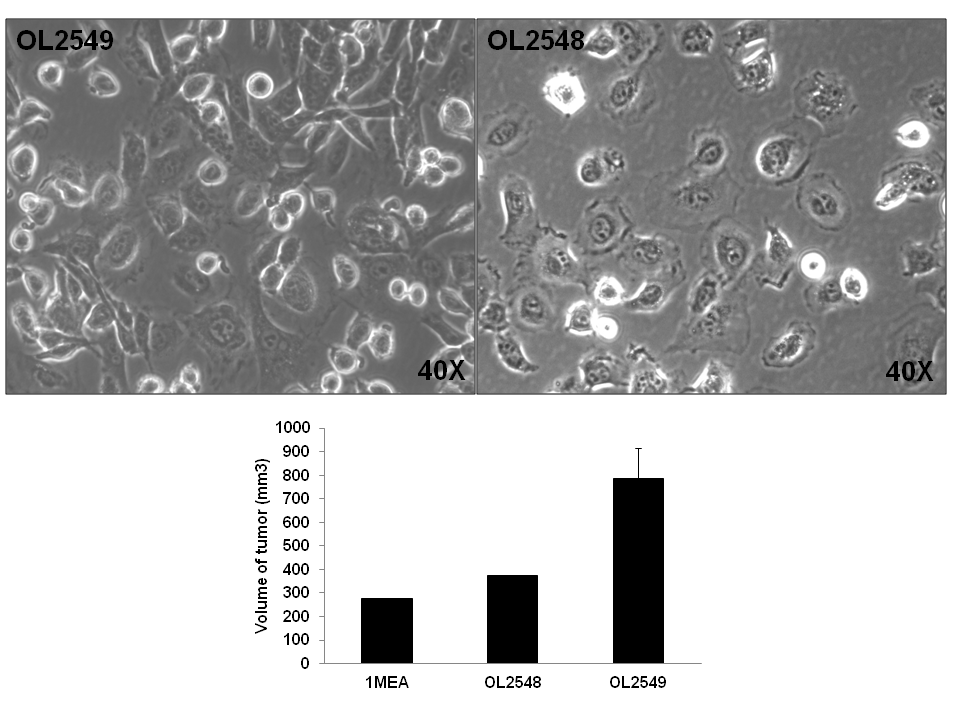

Supplement: Figure S1 — OL2549 and OL2548 are novel CTC lines. Top. One million BNL 1ME A.7R.1 cells were implanted directly into the liver of separate Balb/c mice via survival surgery as described in the “materials and methods” section. The Balb/c mice were humanely euthanized when clinical evidence of tumor development was observed. Isolation of CTCs was performed as described in the “materials and methods” section. Novel CTC lines designated OL2549 and OL2548 were established. They are still viable after multiple passages. Phase contrast images at ×40 magnification (objective lens). Bottom. 1×10^6^ BNL 1ME A.7R.1 or OL2548 or OL2549 cells were subcutaneously implanted into 5 separate Balb/c mice. Tumors from OL2548 and OL2549 cells had greater volume than those from BNL 1ME A.7R.1 cells. (TIF) [file pone.0063765.s001.tif]

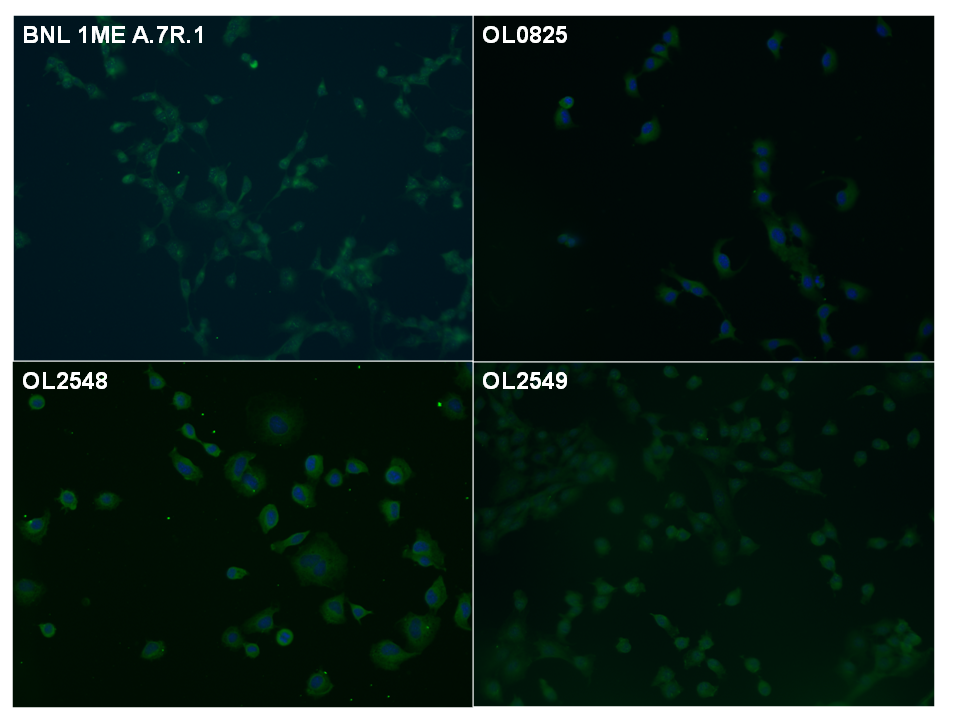

Supplement: Figure S3 — CTC lines and BNL 1ME A.7R.1 cells all express specific marker of hepatocytes. Images of BNL 1ME A.7R.1, OL0825, OL2548 and OL2549 cells specifically stained for expression of the hepatocyte-specific marker CREB3L3. Image magnification was ×20 objective. Green coloration represents CREB3L3 expression and blue coloration represents DAPI-stained nuclei. (TIF) [file pone.0063765.s003.tif]

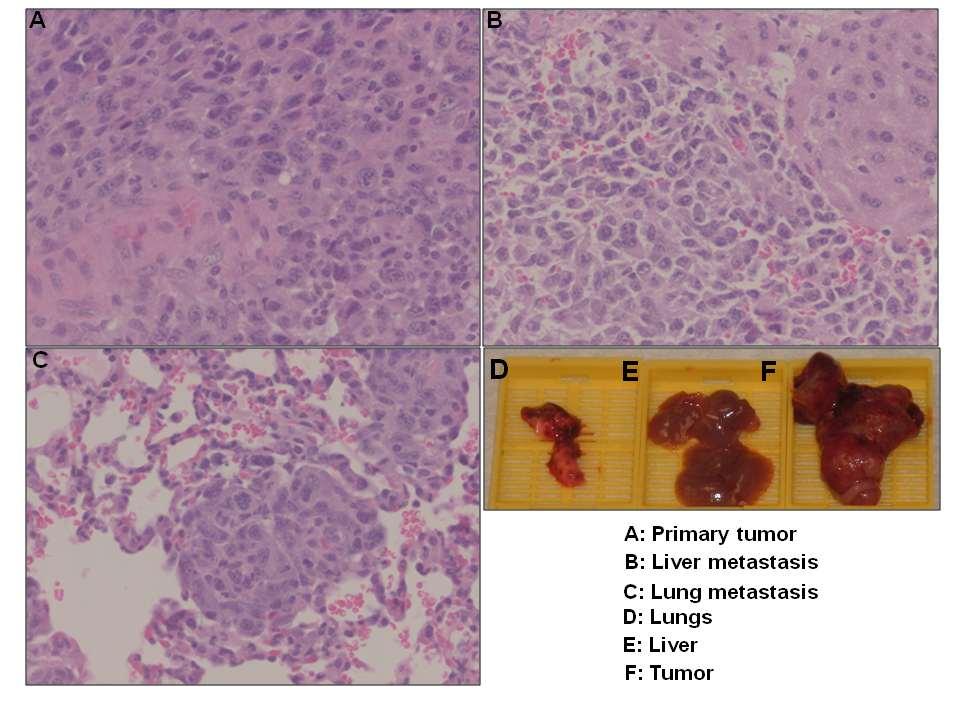

Supplement: Figure S4 — Circulating tumor cells have increased metastatic potential. CTCs were isolated from a Balb/c mouse that developed tumor from implantation of BNL 1ME A.7R.1 cells using the method described in the “materials and methods” section. Isolated CTCs were washed in PBS, suspended in PBS and implanted subcutaneously into the left flank of another Balb/c mouse. Tumor developed in the left flank at site of implantation (F). Macrometastasis was observed in the lungs (D) and liver (E). Hematoxylin and eosin staining of formalin-fixed paraffin-embedded sections revealed histologic evidence of primary tumor at left flank mass (A), metastatic tumor in liver (B) and lungs (C). Bright field images were taken at ×40 magnification (objective lens). (TIF) [file pone.0063765.s004.tif]

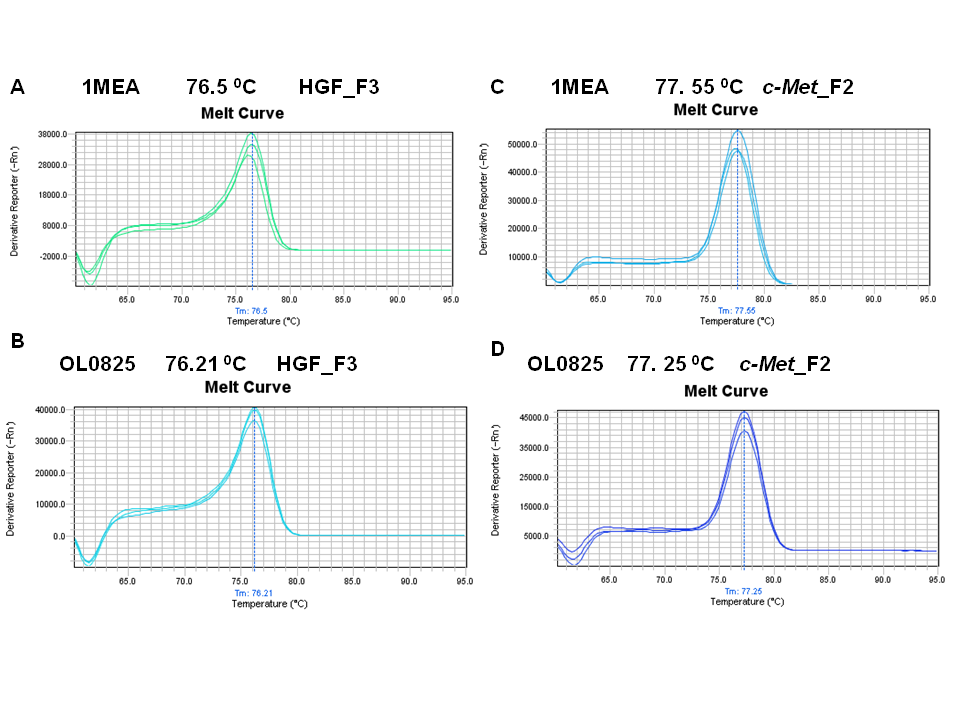

Supplement: Figure S6 — High resolution melt analysis of HGF and c-Met promoters. Melt analysis of the murine HGF promoter and mouse c-Met promoter using HGF_F3 and c-Met_F2 primers indicates that there is a temperature difference between the PCR products from BNL 1ME A.7R.1 cells and OL0825 cells. HGF products from BNL 1ME A.7R.1 cells are 76.5°C (A) whereas products from OL0825 cells have a melt temperature 76.21°C (B) which indicates that OL0825 cells have less methylation than BNL 1ME A.7R.1 cells. c-Met products from BNL 1ME A.7R.1 cells are 77.55°C (C) and products from OL0825 cells are 77.25°C (D) indicating that OL0825 cells have less methylation than 1MEA cells. (TIF) [file pone.0063765.s006.tif]

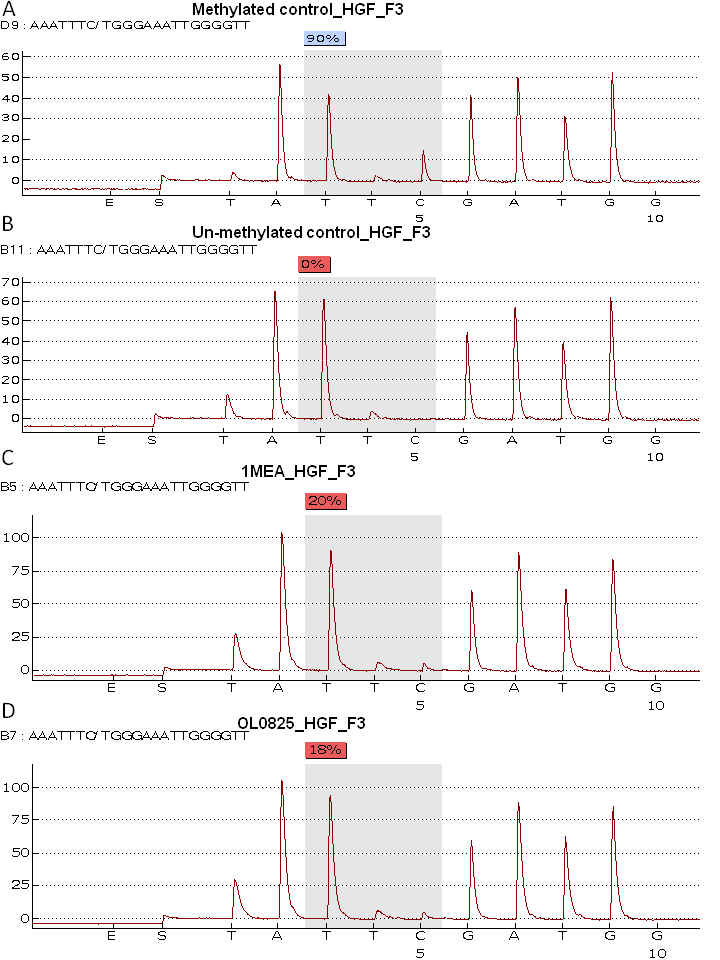

Supplement: Figure S8 — Pyrosequencing analysis of HGF DNA promoter region at first CpG site. The data shows only 2% reduction in DNA methylation at this CpG site between BNL 1ME A.7R.1 and OL0825 cells. (TIF) [file pone.0063765.s008.tif]

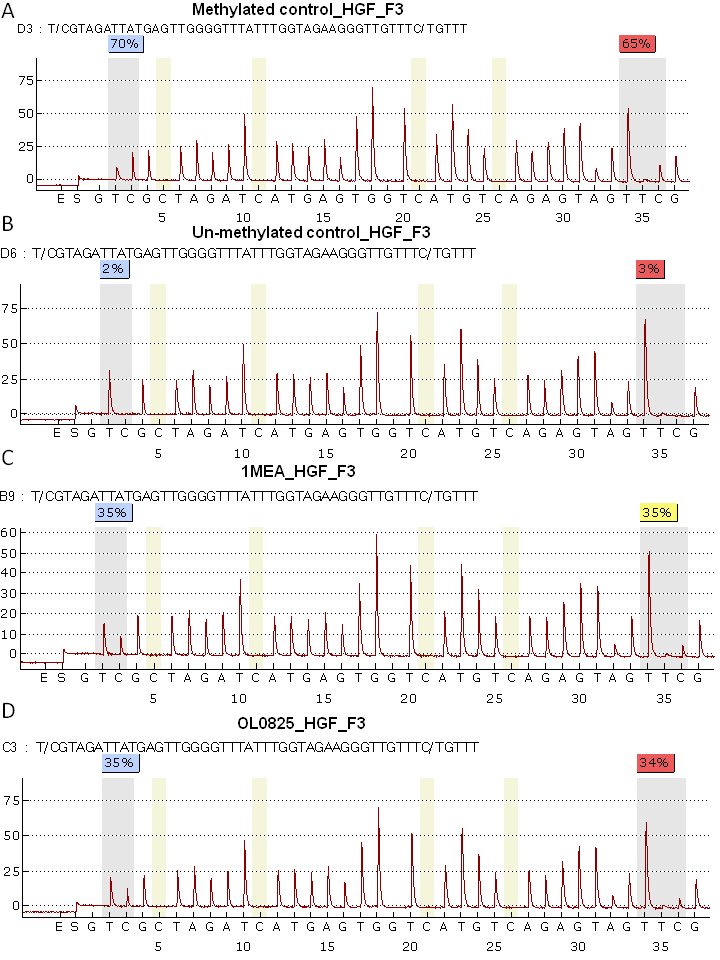

Supplement: Figure S9 — Pyrosequencing analysis of HGF DNA promoter region at two other CpG sites. The data shows only 1% reduction in DNA methylation at one of the two CpG sites between BNL 1ME A.7R.1 and OL0825 cells. (TIF) [file pone.0063765.s009.tif]

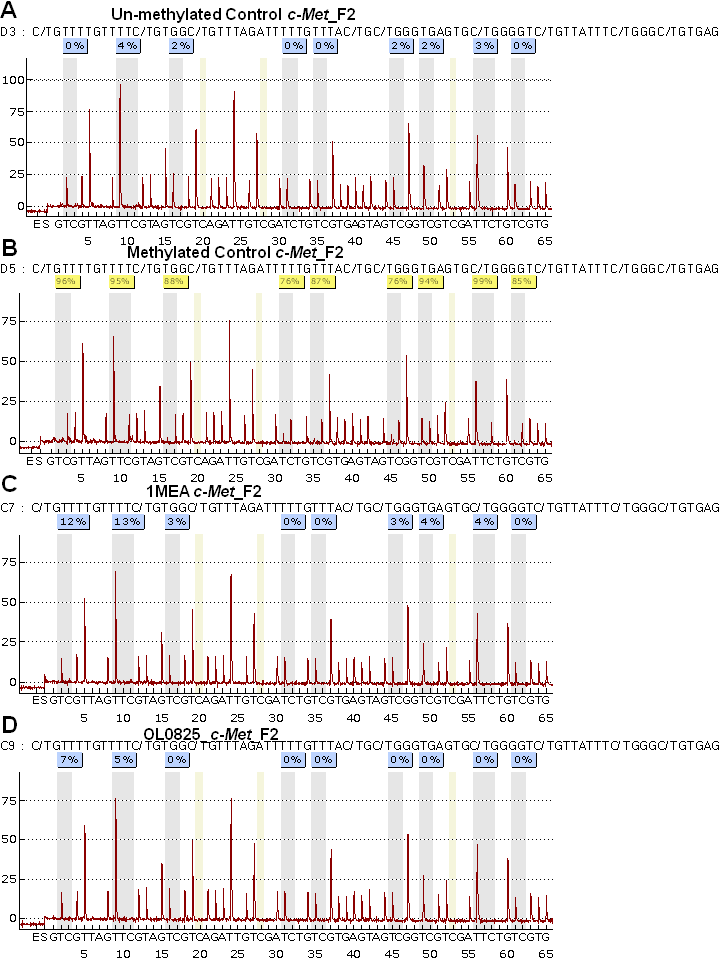

Supplement: Figure S10 — Pyrosequencing analysis of c-Met promoter region (9 CpG sites). The data shows clear reduction in DNA methylation at 6 of 9 CpG sites between BNL 1ME A.7R.1 and OL0825 cells. (TIF) [file pone.0063765.s010.tif]
