# Supplementary material for: Epigenetic Upregulation of HGF and c-Met Drives Metastasis in Hepatocellular Carcinoma
Source: PLoS One. 2013 May 28;8(5):e63765. doi: 10.1371/journal.pone.0063765 (PMC3665785; doi:10.1371/journal.pone.0063765)
Supplement: Table S1 — Primers for a DNA segment of the mouse β-globin gene. (DOCX) [file pone.0063765.s011.docx]

**Table S1** – Primers for a DNA segment of the mouse β-globin gene.

| Primer | 5′ to 3′ | Species | Position | Location | Accession number |
| --- | --- | --- | --- | --- | --- |
| c-globin-F | cct gtg ggg aaa ggt gaa c | M, R | 2759–2777 | Exon 1 | [J00413](http://www.ncbi.nlm.nih.gov/nuccore/193793) and [X06701](http://www.ncbi.nlm.nih.gov/nuccore/56251) |
| glo-mus-R | ata cca gat acc tgc agg ctt at | M | 3735–3757 | Intron 2 | [J00413](http://www.ncbi.nlm.nih.gov/nuccore/193793) |
