# Supplementary material for: Epigenetic Upregulation of HGF and c-Met Drives Metastasis in Hepatocellular Carcinoma
Source: PLoS One. 2013 May 28;8(5):e63765. doi: 10.1371/journal.pone.0063765 (PMC3665785; doi:10.1371/journal.pone.0063765)
Supplement: Table S2 — Primers and probes for pyrosequencing. (DOCX) [file pone.0063765.s012.docx]

**Table S2** – Primers and probes for pyrosequencing.

| **Primer Name** | **Primer Sequence** |
| --- | --- |
|  |  |
| *c-Met*_F2 | 5' CGTTTATGTATTTTTAATATTGTTTGT 3' |
| *c-Met*_R2 | 5' CGAAACTACAACCTCTCTCA 3' |
| *c-Met*_R2_probe | 5' *CGAAACTACAACCTCTCTCA 3' |
| *c-Met*_F2_SEQ | 5' TATTGTTTGTGATAATGAG 3' |
|  |  |
| HGF_F3 | 5' TTGTTTTTATTGTTTTTAAAATTT 3' |
| HGF_R3 | 5' AAATACATATATTTACATATCTATCTAAA 3' |
| HGF_R3_PROBE | 5'*AAATACATATATTTACATATCTATCTAAA 3' |
| HGF_F3_SEQ1 | 5' TGTTTTTATTGTTTTTA 3' |
| HGF_F3_SEQ2 | 5' TTTTTTTAGTATTGTAAGTT 3' |
|  |  |
| * denotes 5' biotinylation and HPLC purified primer | |
