# Supplementary material for: Epigenetic Upregulation of HGF and c-Met Drives Metastasis in Hepatocellular Carcinoma
Source: PLoS One. 2013 May 28;8(5):e63765. doi: 10.1371/journal.pone.0063765 (PMC3665785; doi:10.1371/journal.pone.0063765)
Supplement: Table S3 — Primers and probes for c-Met and HGF promoter analysis. (DOCX) [file pone.0063765.s013.docx]

**Table S3** – Primers and probes for c-Met and HGF promoter analysis.

| **Primer Name** | **Primer Sequence** |
| --- | --- |
|  |  |
| c-Met_F1 | 5' CGAATTAGTTTTTTTGATAAT 3' |
| c-Met_R1 | 5' CGATTACATAAACAAATAACAACTAAC 3' |
| c-Met_F2 | 5' CGTTTATGTATTTTTAATATTGTTTGT 3' |
| c-Met_R2 | 5' CGAAACTACAACCTCTCTCA 3' |
| c-Met_R2a | 5' CCAACGCAAAAAAAAATCAATAA 3' |
| c-Met_F3 | 5' CGTATAGTTGGGGAGAGGGAAATTT 3' |
| c-Met_R3 | 5' CGAAATAAAAACTAACACCACCCTTAAA 3' |
| c-Met_F4 | 5' GATCGTTGGGGATAAATTT 3' |
| c-Met_R4 | 5' TTGTTGTTCGGTTGAGTTA 3' |
| c-Met_F5 | 5' CGGGTGATTTTGTGTGG 3' |
| c-Met_R5 | 5' TCAACCGACTAAAAACCACC 3' |
| c-Met_F6 | 5' TCGGGTATTTTAAGGTATAAATTTT 3' |
| c-Met_R6 | 5' CGACCCCATACCTTCAA 3' |
| c-Met_F7 | 5' CGTTTTAGAAGGAGTGTTGTT 3' |
| c-Met_R7 | 5' CGATCCCTTCTATAAAAACTAAA 3' |
| c-Met_F8 | 5' CGGTAAGGTTTGAGTTTAGTT 3' |
| c-Met_R8 | 5' CGTATATTTACCAAAAATAAAAAA 3' |
| c-Met_R2_probe | 5' *CGAAACTACAACCTCTCTCA 3' |
| c-Met_F2_SEQ | 5' TATTGTTTGTGATAATGAG 3' |
|  |  |
| HGF_F1 | 5' GTGTGTGTAGGAGAAAAGAGGTT 3' |
| HGF_R1 | 5' AATCTTTCTAACCTTATATACAATTACACATA 3' |
| HGF_F2 | 5' GGTTTGAAAATTAGGGTGTTTTT 3' |
| HGF_R2 | 5' CAAAACCCAAATAAAAAAAAAACA 3' |
| HGF_F3 | 5' TTGTTTTTATTGTTTTTAAAATTT 3' |
| HGF_R3 | 5' AAATACATATATTTACATATCTATCTAAA 3' |
| HGF_F4 | 5' TATGAGTTGGGGTTTATTT 3' |
| HGF_R4 | 5' CTAAAACTCCAAATCCTTATAA 3' |
| HGF_R4a | 5' CCAAAAAAAAACTTACAAAA 3' |
| HGF_PROBE | 5'*AAATACATATATTTACATATCTATCTAAA 3' |
| HGF_F3_SEQ1 | 5' TGTTTTTATTGTTTTTA 3' |
| HGF_F3_SEQ2 | 5' TTTTTTTAGTATTGTAAGTT 3' |
|  |  |
| * denotes 5' biotinylation and HPLC purified primer | |
